# Coordinated Loss and Acquisition of NK Cell Surface Markers Accompanied by Generalized Cytokine Dysregulation in COVID-19

**DOI:** 10.3390/ijms24031996

**Published:** 2023-01-19

**Authors:** Maria O. Ustiuzhanina, Julia D. Vavilova, Anna A. Boyko, Maria A. Streltsova, Sofya A. Kust, Leonid M. Kanevskiy, Alexander M. Sapozhnikov, Rustam N. Iskhakov, Ekaterina O. Gubernatorova, Marina S. Drutskaya, Mikhail V. Bychinin, Oksana A. Zhukova, Oksana N. Novikova, Anna G. Sotnikova, Gaukhar M. Yusubalieva, Vladimir P. Baklaushev, Elena I. Kovalenko

**Affiliations:** 1 Shemyakin & Ovchinnikov Institute of Bioorganic Chemistry, Russian Academy of Sciences, 117997 Moscow, Russia; 2 Center of Life Sciences, Skolkovo Institute of Science and Technology, 121205 Moscow, Russia; 3 Center for Precision Genome Editing and Genetic Technologies for Biomedicine, Engelhardt Institute of Molecular Biology, Russian Academy of Sciences, 119991 Moscow, Russia; 4 Division of Immunobiology and Biomedicine, Center of Genetics and Life Sciences, Sirius University of Science and Technology, 354340 Federal Territory Sirius, Russia; 5 Federal Research and Clinical Center of Specialized Medical Care and Medical Technologies FMBA of Russia, 115682 Moscow, Russia

**Keywords:** COVID-19, SARS-CoV-2, NK cells, phenotype, adaptive-like NK cells, cytokines, coordinative response

## Abstract

Coronavirus disease 2019 (COVID-19), caused by the SARS-CoV-2 virus, is accompanied by a dysregulated immune response. In particular, NK cells, involved in the antiviral response, are affected by the infection. This study aimed to investigate circulating NK cells with a focus on their activation, depletion, changes in the surface expression of key receptors, and functional activity during COVID-19, among intensive care unit (ICU) patients, moderately ill patients, and convalescents (CCP). Our data confirmed that NK cell activation in patients with COVID-19 is accompanied by changes in circulating cytokines. The progression of COVID-19 was associated with a coordinated decrease in the proportion of NKG2D^+^ and CD16^+^ NK cells, and an increase in PD-1, which indicated their exhaustion. A higher content of NKG2D^+^ NK cells distinguished surviving patients from non-survivors in the ICU group. NK cell exhaustion in ICU patients was additionally confirmed by a strong negative correlation of PD-1 and natural cytotoxicity levels. In moderately ill patients and convalescents, correlations were found between the levels of CD57, NKG2C, and NKp30, which may indicate the formation of adaptive NK cells. A reduced NKp30 level was observed in patients with a lethal outcome. Altogether, the phenotypic changes in circulating NK cells of COVID-19 patients suggest that the intense activation of NK cells during SARS-CoV-2 infection, most likely induced by cytokines, is accompanied by NK cell exhaustion, the extent of which may be critical for the disease outcome.

## 1. Introduction

Novel coronavirus disease 2019 (COVID-19), caused by severe acute respiratory syndrome coronavirus 2 (SARS-CoV-2), has rapidly spread all over the world; the number of cases is more than half a billion, and more than 6 million people have already died [1,2]. The symptoms vary from asymptomatic to severe, which can be accompanied by acute respiratory distress syndrome (ARDS) [3]. The variable clinical outcome in infected individuals is due to the complex interaction of the virus with the immune system. NK cells, effectors of innate immunity, along with CD8^+^ T cells, mediators of adaptive immunity, are important components of the antiviral immune response [4]. NK cells exhibit a rapid effector response with the ability to directly kill infected cells and perform antibody-dependent cytotoxicity. In addition, NK cells are endowed with immunomodulatory functions and are able to regulate the innate and adaptive immune responses through the secretion of cytokines and interaction with other immune cells.

SARS-CoV-2 remodels the NK cell phenotype and function. For their part, NK cells may contribute to the defense against SARS-CoV-2 infection and play an important role in the COVID-19 outcome [5,6]. Viral load was shown to decrease more rapidly with normal but not depleted levels of blood NK cells, and no such correlation was found for T or B cells [7]. Nevertheless, the question of the predictive significance of NK cell characteristics in COVID-19 remains relevant.

Over the past two years, most reports on COVID-19 have highlighted lymphopenia as the main feature reflecting the severity of COVID-19 [8]. A decrease in both percentages and absolute counts of NK cells in the peripheral human blood was repeatedly reported [9,10,11,12]. At the same time, the main NK cell subsets in patients with COVID-19 undergo qualitative changes. In line with this, scRNA-seq analysis showed that CD56^dim^ NK cells were depleted mainly in ICU patients, while CD56^bright^ NK cells were significantly decreased in patients with COVID-19, regardless of severity [13]. However, after recovery from a severe form of COVID-19, the number of NK cells was quickly restored [14].

NK cells are involved in cytokine production in response to IFN-α in early SARS-CoV-2 infection [15]. It was shown that NK cells from patients with COVID-19 have an activated phenotype. Specifically, HLA-DR expression was significantly increased in the NK cells of COVID-19 patients [10]. A higher level of CD107a expression and IFN-γ production in NK cells was reported for severe SARS-CoV-2 patients compared to moderate ones [16]. However, several findings indicate that SARS-CoV-2 infection can promote an exhausted NK cell phenotype [15,17]. The PD-1 inhibitory receptor, a checkpoint molecule with immunoregulatory properties, is increased in T lymphocytes in COVID-19 [18]. This molecule is a less common exhaustion marker for NK cells, although higher PD-1 expression was associated with reduced IFN-γ secretion and a lower degranulation degree in this cell subset [19].

NKG2A and KIR2DL2/DL3 are the most common inhibitory NK cell receptors, which play a role in distinguishing between self and non-self and in “licensing” NK cells for effective killing. Heterodimer CD94/NKG2A interacts with the HLA-E molecule capable of presenting host or viral peptides to NK cells [20]. An increase in the NKG2A-expressing NK cell subset in COVID-19 patients has been reported earlier [1], whereas other studies have not supported this fact [21,22]. KIR2DL2 and KIR2DL3 interact with HLA-C molecules [23]. Notably, the combination of KIR2DL2 and HLA-C1 in one genotype was shown to be associated with an increased risk of SARS-CoV-2 infection [24]. In general, surface expression of KIRs indicates a more differentiated state of NK cells.

NKG2D is one of the key NK-cell-activating receptors, which plays a role in both anticancer and antiviral immunity [25]. NKG2D interacts with stress molecules on the surfaces of infected cells, such as MICA, MICB, and ULBP1-6 [26]. Interaction of the soluble forms of these molecules with NKG2D results in a decreased NKG2D surface level and, as a result, decreased natural cytotoxicity. Low NKG2D expression on the surfaces of NK cells was reported in COVID-19 [27]. FcγRIII (CD16) is another extremely important activating NK cell receptor responsible for antibody-dependent cell-mediated cytotoxicity (ADCC) [28], which was also shown to be decreased in COVID-19 [29]. These findings evidence that the dysfunction of NK cells may develop during COVID-19. At the same time, recent studies point to the great importance of ADCC in the control of viral infection in COVID-19 [16,30].

Recently, NK cells have been shown to specifically respond to certain pathogens with the formation of a pool of differentiated cells, which undergo epigenetic remodeling. Cytomegalovirus infection (HCMV) is widespread in the human population and leads to a stable change in the NK cell repertoire [31]. HCMV-associated adaptive NK cells are characterized by a terminally differentiated phenotype (NKG2A^−^KIR^+^CD57^+^), reduced expression of PLZF transcription factor, Syk signaling molecule, FcεRIγ adapter chain, and NKp46 and NKp30 receptors [32]. Adaptive NK cells expand upon HCMV infection and often, although not always, express the NKG2C receptor [33]. The interaction of the CD94/NKG2C heterodimer and its cellular ligand HLA-E mediates the release of proinflammatory effector molecules, in addition to cytotoxic responses of NK cells against the virus-infected cells [34]. According to the literature, the pools of NKG2C^+^ NK cells and FcεRIγ-negative NK cells partially overlap [35]. There are contradictory data on the role of adaptive/adaptive-like NK cells in the control of SARS-CoV-2 infection and the development of COVID-19. Some studies incline towards a negative role of the adaptive-like NK cells. For example, the increased content of highly differentiated NK cells with a reduced level of FcεRIγ expression [27] or an expanded NKG2C-expressing NK cell subset were detected [10]. On the other hand, it has been previously shown that the absence of a gene encoding the activating NKG2C receptor in NK cells is a risk factor for severe COVID-19 disease [36].

Overall, the accumulated data on NK cells in COVID-19 disease are incomplete and in some ways contradictory. An additional difficulty in studying the immune response to SARS-CoV-2 is the diversity of viral strains that have emerged during the pandemic. Compared to the less severe Omicron strain, the Wuhan strain, which dominated at the early stages of the pandemic, caused more severe cases and deaths. The lack of immunological memory against SARS-CoV-2, formed during the earlier history of human contact with this virus, may have contributed to this fact. Our study was performed on samples from moderate and severe patients with COVID-19 collected during the first wave of the pandemic (May–July 2020) at the Federal Research and Clinical Center for Specialized Types of Medical Care and Medical Technologies (FMBA), Moscow, Russia. Groups of convalescents and healthy donors were also examined. The phenotype of circulating NK cells was analyzed and included the surface levels of activation markers (HLA-DR and PD-1), key inhibitory and activating receptors (NKG2A, KIR2DL2/DL3, NKG2D, CD16), a marker of terminal differentiation (CD57), and markers associated with adaptive-like NK cell phenotypes (NKG2C, NKp30). In addition, granzyme B intracellular levels and degranulation in response to K562 cells were measured. We also compared the different subpopulation proportions between non-survivors and recovered patients in the severe group. Correlation analysis of the above-mentioned marker expression levels was performed.

## 2. Results

### 2.1. Severe COVID-19 Is Accompanied by the Depletion of Circulating Lymphocytes and a Decrease in the Content and Viability of NK Cells

**Figure 1:**
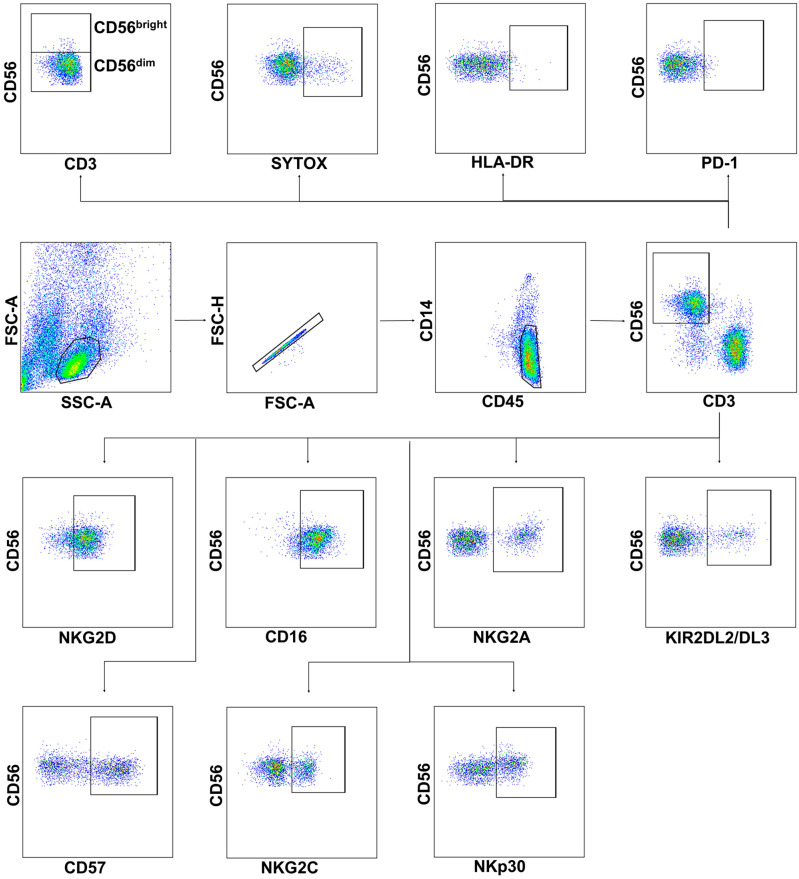
The gating strategy for cytometric analysis of NK cells in PBMC samples. Surface expression of PD-1, HLA-DR, NKG2D, CD16, NKG2A, KIR2DL2/DL3, CD57, NKG2C, and NKp30 was analyzed in NK cells by flow cytometry after staining with fluorescent-labeled specific monoclonal antibodies. NK cells were defined as CD3−CD56+ cells in CD45highCD14− cells in the FSC-SSC lymphocyte gate; CD56bright and CD56dim NK cells were determined. SytoxBlue staining was used to detect dying cells.

The percentage of lymphocytes (CD45^high^CD14^−^) and, in particular, NK cells and their subsets was measured by flow cytometry in the PBMC samples from COVID-19 patients at the intensive care unit (ICU patients), patients with COVID-19 of moderate severity, COVID-19 convalescent plasma donors (CCP donors), and healthy donors. The gating strategy for determination of the lymphocytes, NK cells, and surface marker levels is presented in Figure 1. 

**Figure 2:**
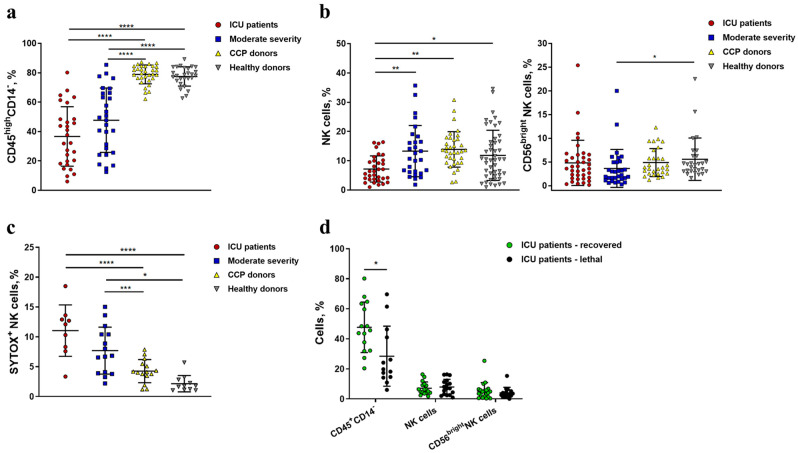
Peripheral blood levels and viability of lymphocytes from COVID-19 patients, convalescent patients, and healthy donors. (**a**) CD45^high^CD14^−^ cell percentage in PBMC was assessed in the following groups: ICU patients (n = 28), moderate-severity patients (n = 28), CCP donors (n = 31), and healthy donors (n = 27). (**b**) NK cell percentage among CD45^high^CD14^−^ cells and CD56^bright^ NK cell percentage among all NK cells in blood samples from ICU patients (n = 34), moderate-severity patients (n = 31), CCP donors (n = 31), and healthy donors (n = 48). (**c**) Percentage of dead cells measured in blood samples from ICU patients (n = 9), moderate-severity patients (n = 15), CCP donors (n = 15), and healthy donors (n = 15). (**d**) Comparative analysis of CD45^high^CD14^−^ cell, NK cell, and CD56^bright^ NK cell levels in the recovered and lethal patients from the ICU group. Data are presented as individual values with mean (±SD). * *p* < 0.05, ** *p* < 0.01, *** *p* < 0.001, **** *p* < 0.0001.

In concordance with other research groups [9,10,11,12,37], we registered a decrease in the lymphocyte pool in ICU patients and moderate-severity patients compared to CCP donors and healthy individuals (Figure 2a). A critically low percentage of CD45^high^CD14^−^ cells was detected among non-survivors from the ICU group as compared to recovered patients, which confirms the prognostic value of this parameter (Figure 2d). NK cells (defined as CD56^+^CD3^−^ cells) were significantly and vastly decreased in ICU patients compared to all other groups (Figure 2b), with no differences between recovered and lethal patients (Figure 2d). A higher percentage of SytoxBlue-Dead-Cell-stain-positive cells was found in the NK cell population in the ICU patient group compared to moderate patients, CCP, and healthy donors (Figure 2c), indicating a decrease in the viability of these cells during COVID-19 infection, which possibly contributed to the observed lymphocyte depletion.The proportion of less differentiated CD56^bright^ NK cells was reduced and, accordingly, the proportion of more differentiated CD56^dim^ NK cells was increased in patients with COVID-19 of moderate severity as compared to healthy donors. However, such a pattern was not detected in the ICU patients, as the proportion of CD56^bright^ NK cells in this group was quite variable (Figure 2b). Survivors did not differ in the proportion of CD56^bright^ NK cells from non-survivors in the same group (Figure 2d).

**Figure 3:**
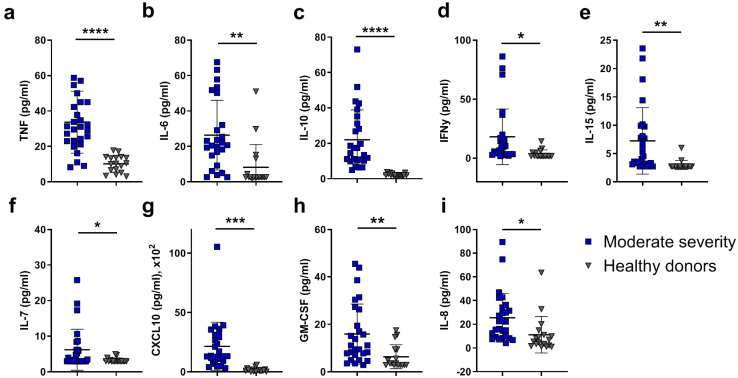
Systemic production of cytokines and chemokines in the blood of patients with moderate-severity disease indicates the activation of a broad immune response. Concentration of (**a**) TNF, (**b**) IL-6, (**c**) IL-10, (**d**) IFNγ, (**e**) IL-15, (**f**) IL-7, (**g**) CXCL10, (**h**) GM-CSF, (**i**) IL-8 in pg/mL in the serum of healthy donors and moderate-severity patients. Data are presented as individual values with mean (±SD). * *p* < 0.05, ** *p* < 0.01, *** *p* < 0.001, **** *p* < 0.0001.

In order to assess the inflammatory context, which may directly affect blood cell numbers and functions, systemic cytokine profiling was performed on serum samples of patients with moderate disease and compared to the healthy control group (Figure 3 and Figure S1). A significant reduction in circulating lymphocytes, including NK cells, was accompanied by an increase in the serum levels of inflammatory mediators in patients with moderate COVID-19 as compared to healthy donors. The inflammatory response was characterized by upregulation of the typical “cytokine storm” markers such as TNF (Figure 3a), IL-6 (Figure 3b), IL-10 (Figure 3c), and IFN-γ (Figure 3d), in agreement with previously published studies [38,39,40]. Increased concentrations of IFN-γ, IL-15 (Figure 3e), and IL-7 (Figure 3f) may contribute to the activation and survival, as well as exhaustion, of lymphocytes, including NK cells. Low numbers of NK cells detected in the peripheral blood of patients with COVID-19 may also be due to the recruitment of pathogenic NK cells to the lungs in response to high levels of CXCL10 (Figure 3g), accompanied by their activation and the induction of IFN-γ production. On the other hand, both CXCL10 [41] and GM-CSF (Figure 3h) drive myeloid cell recruitment and activation, including neutrophils, as indicated by the high levels of IL-8 (Figure 3i) [42], further contributing to the inflammatory response in the acute phase of SARS-CoV-2 infection.

### 2.2. NK Cells in COVID-19 Show an Activated Profile with Signs of Exhaustion

**Figure 4:**
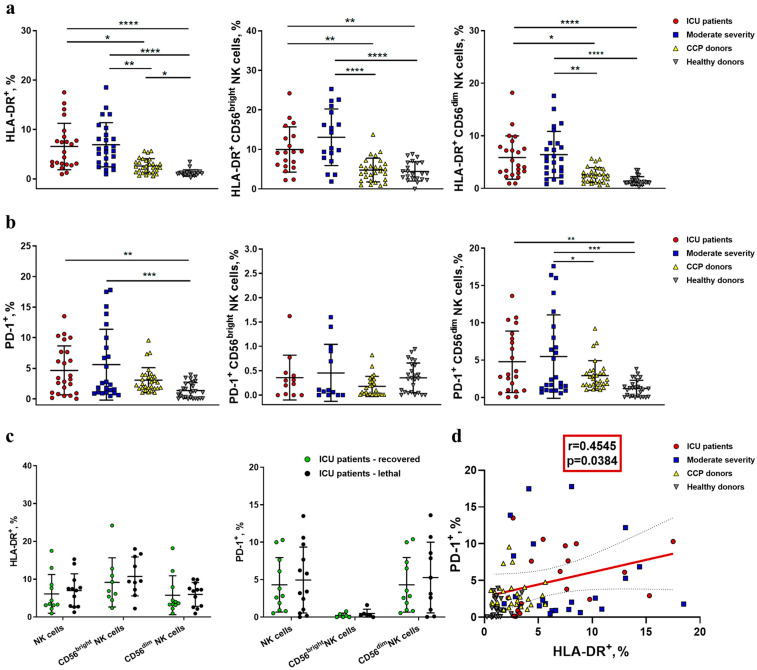
Analysis of activation and exhaustion markers in NK cells from COVID-19 patients, convalescent patients, and healthy donors. (**a**) The proportion of HLA-DR^+^ cells among NK cells and CD56^bright^ and CD56^dim^ subsets in the following comparison groups: ICU patients (n = 23), moderate-severity patients (n = 25), CCP donors (n = 29), and healthy donors (n = 24). (**b**) The proportion of PD-1^+^ cells among NK cells and CD56^bright^ and CD56^dim^ subsets in the following comparison groups: ICU patients (n = 31), moderate-severity patients (n = 25), CCP donors (n = 29), and healthy donors (n = 28). (**c**) Comparative analysis of HLA-DR^+^ and PD-1^+^ cell percentages among NK cells and CD56^bright^ and CD56^dim^ subsets in recovered and lethal ICU patients from ICU group. (**d**) Spearman correlation between HLA-DR and PD-1 expression in all donor groups. (**a**–**c**) Data are presented as individual values with mean (±SD). * *p* < 0.05, ** *p* < 0.01, *** *p* < 0.001, **** *p* < 0.0001.

To assess the functional profile of NK cells during COVID-19 infection, we analyzed the expression of one of the human leukocyte antigen class II molecules—HLA-DR, a common marker of lymphocyte activation—in all NK cells and in the less differentiated CD56^bright^ subset and more differentiated CD56^dim^ subset. We also analyzed the expression of checkpoint inhibitory receptor PD-1, which appears on the NK cell surface during prolonged activation and can be regarded as a marker of cell exhaustion (Figure 4) [19].

The percentage of the activated HLA-DR^+^ subset was higher in NK cells from both ICU and moderate-severity COVID-19 patients, compared to the CCP donor and healthy donor groups (Figure 4a and Figure S2). In CD56^bright^ NK cells, the COVID-19-induced increase in the HLA-DR expression level in the moderate-severity group was even more significant compared to the control group than in the ICU group (Figure 4a). No significant difference was shown in the HLA-DR expression level in NK cells between recovered patients and patients with a lethal outcome in the ICU group (Figure 4c).

An increased percentage of PD-1-expressing NK cells was registered in the ICU and moderate-severity patients compared to healthy donors, but only in the more differentiated CD56^dim^ subset (Figure 4b). PD-1 expression had no association with lethality in the ICU patient group (Figure 4c). However, we observed a weak positive correlation between PD-1 and HLA-DR levels in the ICU patient group, which implies some degree of coordination in the acquisition of these markers (Figure 4d).

### 2.3. Reduced Expression of NKG2D and CD16 Receptors Is Observed on the Surface of NK Cells during COVID-19

**Figure 5:**
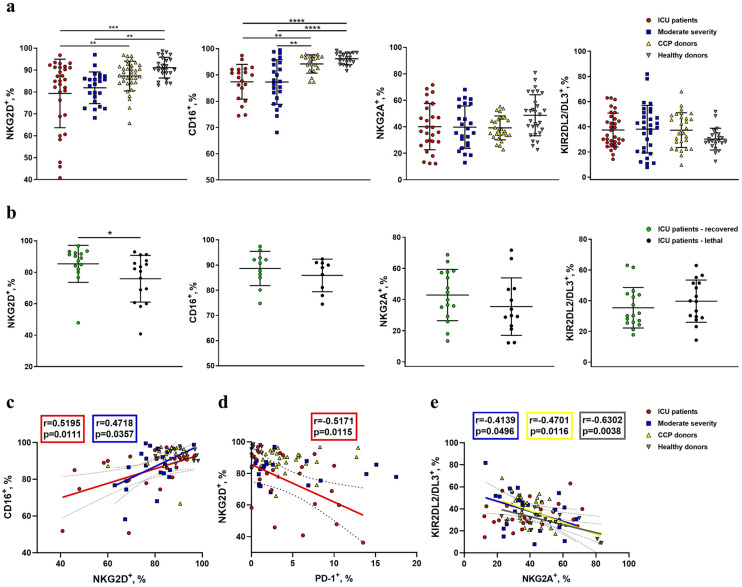
Analysis of NKG2D, CD16, NKG2A, and KIR3DL2/DL3 expression in NK cells from COVID-19 patients, convalescent patients, and healthy donors. (**a**) The proportion of NKG2D^+^, CD16^+^ NKG2A^+^, and KIR3DL2/DL3^+^ NK cells measured in the following comparison groups: ICU patients (n = 32, n = 20, n = 27, and n = 27, respectively), moderate-severity patients (n = 23, n = 23, n = 24, and n = 24, respectively), CCP donors (n = 35, n = 20, n = 27, and n = 27, respectively), and healthy donors (n = 27, n = 21, n = 29, and n = 29, respectively). (**b**) Comparative analysis of NKG2D^+^, CD16^+^, NKG2A^+^, and KIR3DL2/DL3^+^ NK cells in recovered and lethal ICU patients from the ICU group. (**c**) Spearman correlation between NKG2D^+^ and CD16^+^ NK cell levels in all donor groups. (**d**) Pearson correlation between NKG2D^+^ and PD-1^+^ NK cell levels in all donor groups. (**e**) Pearson correlation between NKG2A^+^ and KIR3DL2/DL3^+^ NK cell levels in all donor groups. (**a**,**b**) Data are presented as individual values with mean (±SD). * *p* < 0.05, ** *p* < 0.01, *** *p* < 0.001, **** *p* < 0.0001.

The surface expression of NK-cell-activating receptors NKG2D and CD16 was analyzed in NK cells (Figure 5 and Figure S2). We observed a significant decrease in NKG2D^+^ cell percentage in NK cells from COVID-19 patients compared to healthy donors, with critically low cases registered in the ICU patient group (Figure 5a). Notably, the percentage of NKG2D^+^ NK cells was lower among non-survivors compared to surviving patients in the ICU group (Figure 5b). The levels of NKG2D-expressing NK cells were comparable among convalescents and healthy donors.

A decrease in NKG2D^+^ NK cells with the progression of COVID-19 was accompanied by a decrease in CD16^+^ NK cell levels (Figure 5a). A significant positive correlation between NKG2D^+^ and CD16^+^ cell percentages was registered for samples obtained from ICU and moderate-severity patients, while no correlation was detected for samples from CCP and healthy donors (Figure 5c). This suggests a coordinated loss of CD16- and NKG2D-expressing NK cells during the COVID-19 immune response. Correlation analysis between NKG2D^+^ and PD-1^+^ NK cell levels from different patient groups revealed a significant negative correlation in the ICU patient group (Figure 5d), supporting the idea of NK cell exhaustion in ICU patients, manifested by the loss of NKG2D and acquisition of PD-1 expression.

Surface expression of inhibitory receptors NKG2A and KIR2DL2/DL3 was also evaluated with regard to NK cell activation profile analysis and COVID-19 progression. Upregulation of NKG2A expression in NK cells from COVID-19 patients has been previously reported and was proposed as an exhaustion factor attenuating NK cell antiviral activity [8,43].

No significant differences in NKG2A^+^ and KIR2DL2/DL3^+^ NK cell percentages were observed among the studied groups (Figure 5a). Moreover, the percentages of both NKG2A^+^ and KIR2DL2/DL3^+^ NK cells were not related to the outcome of the disease in the group of ICU patients (Figure 5b). We established a negative correlation between NKG2A- and KIR2DL2/DL3-expressing NK cell levels in moderate-severity patients, CCP, and healthy donors. Interestingly, ICU patients were the only group in which no correlation was observed (Figure 5e), suggesting dysregulation of these NK cell markers’ expression in severe COVID-19.

### 2.4. Increased NKG2C Expression Accompanies the Formation of Adaptive-Like NK Cells in Moderate COVID-19, While in Severe COVID-19, Lethal Outcome Is Associated with a Decrease in the NKp30^+^ NK Cell Proportion

**Figure 6:**
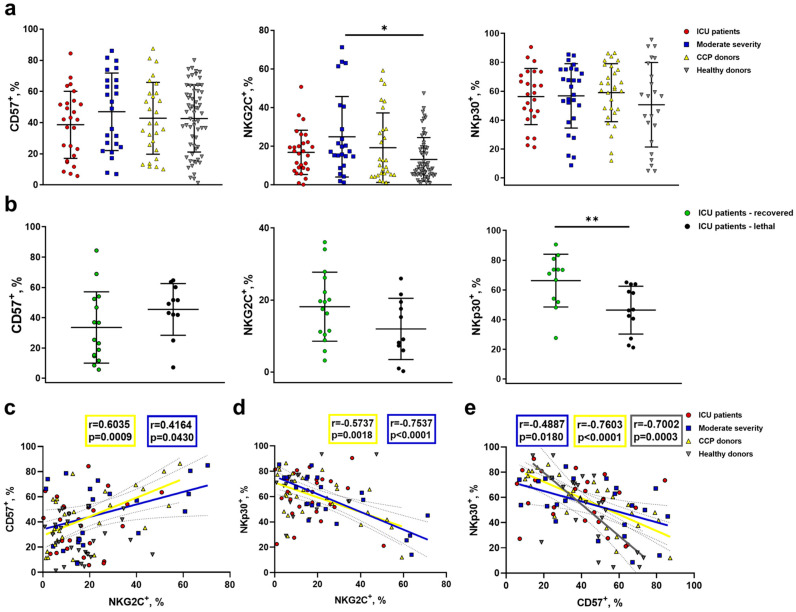
Analysis of NKG2C, CD57, and NKp30 expression in NK cells from COVID-19 patients, convalescent patients, and healthy donors. (**a**) The proportion of CD57^+^, NKG2C^+^, and NKp30^+^ NK cells measured in the following comparison groups: ICU patients (n = 32), moderate-severity patients (n = 24), CCP donors (n = 35), and healthy donors (n = 27 for NKp30, n = 62 for CD57 and NKG2C). (**b**) Comparative analysis of CD57^+^, NKG2C^+^, and NKp30^+^ NK cells in recovered and lethal ICU patients from the ICU group. (**c**) Pearson correlation between the proportions of CD57^+^ and NKG2C^+^ NK cells in all donor groups. (**d**) Pearson correlation between the proportions of NKp30^+^ and NKG2C^+^ NK cells in all donor groups. (**e**) Pearson correlation between the proportions of NKp30^+^ and CD57^+^ NK cells in all donor groups. (**a**,**b**) Data are presented as individual values with mean (±SD). * *p* < 0.05, ** *p* < 0.01.

Since HCMV is widespread in the human population, and in Russia, the abundance varies from 72% to 100% [44], apparently, a significant part of the analyzed patients with COVID-19 initially had adaptive NK cells associated with HCMV in their circulation. Adaptive HCMV-associated NK cells are commonly defined by surface markers CD57, NKG2C, and several others [45]. In addition, adaptive NK cells are characterized by reduced expression of the adapter molecule FcεRγ and a reduced surface level of NKp46 and NKp30 receptors regulated by FcεRγ [33]. In this study, we analyzed changes in the surface expression of CD57, NKG2C and NKp30 in NK cells during COVID-19 (Figure 6 and Figure S2).

Expression of CD57 in NK cells is associated with the high differentiation status of NK lymphocytes and/or cellular senescence [46]. A comparative analysis of the proportion of CD57^+^ NK cells was carried out in patients with varying COVID-19 severity, CCP donors, and healthy individuals. Since the proportion of CD57^+^ NK cells is known to increase with age [47], to minimize the influence of this factor, 34 elderly healthy donors were additionally included in the study (median age: 56 years). However, we found no differences in the percentage of CD57^+^ NK cells among all studied groups (Figure 6a).

The NKG2C receptor expression level in NK cells also increases with age, apparently due to an increase in the HCMV infection’s spread among the population [47]. Thus, we again included in the NKG2C expression analysis the additional group of aged-matched healthy donors. The NKG2C^+^ NK cell percentage was significantly higher in moderate-severity patients compared to healthy individuals (Figure 6a). No significant differences in the percentage of NKp30^+^ NK cells between the analyzed groups were found (Figure 6a). Of note, we observed no increase in NKG2C^+^ NK cell percentage in ICU patients with a lethal outcome compared to ICU survivors (Figure 6b). In moderate-severity patients and CCP donors, we registered a weak but significant positive correlation between CD57^+^ and NKG2C^+^ NK cell levels, which supports coordinated changes in the expression of these markers. Negative correlations were found between NKp30^+^ and NKG2C^+^ NK cell levels, and NKp30^+^ and CD57^+^ NK cell levels, in the same donor groups, favoring the opposite roles of these subsets in the NK cell immune response against COVID-19 in the case of moderate disease or during recovery. No correlations were registered in the ICU group (Figure 6c–e); however, importantly, the NKp30^+^ NK cell percentage was significantly lower in the ICU patients with lethal outcomes compared to the survivors (Figure 6b).

Our data support the idea of the co-expression of NKG2C and CD57 with the coordinated downregulation of NKp30 in NK cells from moderate-severity patients and CCP donors and, thus, the formation of an adaptive-like NK cell subset, whereas, in severe COVID-19 disease, NKG2C, CD57, and NKp30 expression in NK cells seems to be unrelated. Nevertheless, the decreased NKp30 expression level in the ICU group may be considered as a risk factor.

### 2.5. NK Cells Demonstrate an Increased Level of Granzyme B and Decreased K562-Cell-Induced Degranulation in COVID-19

**Figure 7:**
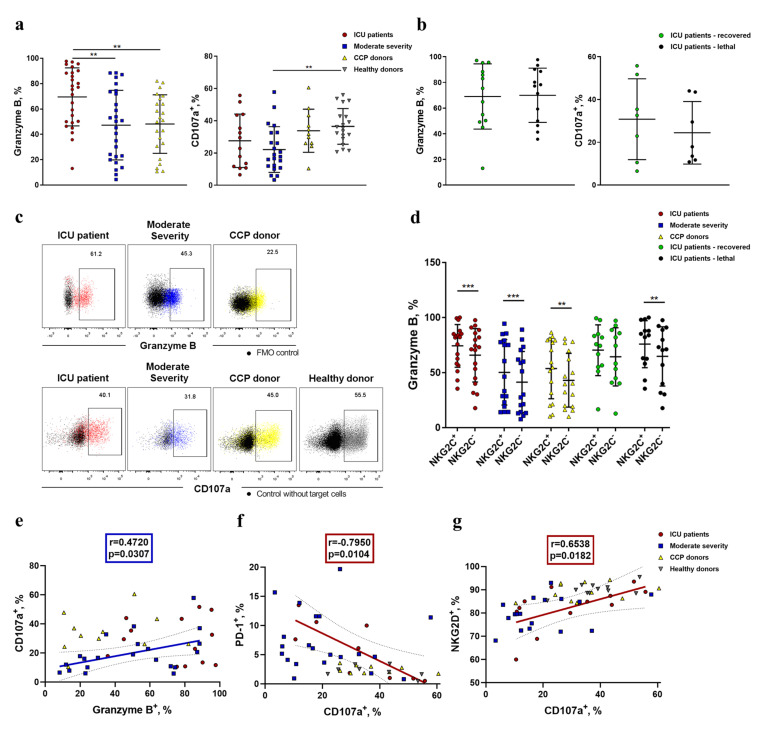
Degranulation activity and granzyme B expression in NK cells from COVID-19 patients, convalescent patients, and healthy donors. (**a**) The proportion of CD107a^+^ and granzyme B^+^ NK cells in the following comparison groups: ICU patients (n = 14 and n = 26, respectively), moderate-severity patients (n = 22 and n = 27, respectively), CCP donors (n = 11 and n = 23, respectively), and healthy donors (n = 18 only for CD107a). (**b**) Comparative analysis of CD107a^+^ and granzyme B^+^ NK cell levels in the recovered and non-recovered patient groups from the ICU. (**c**) Representative dot plots of CD107a and granzyme B expression in NK cells in all studied groups with the respective controls without target cells (K562) or FMO (fluorescence minus one) controls (black), the meaning of other colors is highlighted in the upper part of dot plots. (**d**) Comparative analysis of granzyme B^+^ cell level among NKG2C^+/−^ NK cells measured in the following comparison groups: ICU patients (n = 26), moderate-severity patients (n = 27), CCP donors (n = 23), ICU patients—recovered (n = 12), and ICU patients—non-survivors (n = 13). (**e**) Spearman correlation between the proportions of CD107^+^ and granzyme B^+^ NK cells in the studied groups. (**f**) Pearson correlation between the proportions of CD107^+^ and PD-1^+^ NK cells in the studied groups. (**g**) Spearman correlation between the proportions of CD107^+^ and NKG2D^+^ NK cells in the studied groups. (**a**,**b**,**d**) Data are presented as individual values with mean (±SD). ** *p* < 0.01, *** *p* < 0.001.

Next, to assess the cytotoxic potential of NK cells, we analyzed the intracellular accumulation of granzyme B, a component of cytotoxic NK cell granules [48], in ICU patients, moderate-severity patients, and CCP donors (Figure 7). The highest level of granzyme B was observed in the NK cells of the ICU patients compared with both the moderate-severity group and CCP donors (Figure 7a). No statistically significant differences in granzyme B production were found between the lethal and recovered patients (Figure 7b). Interestingly, NK cells expressing NKG2C contained more granzyme B compared to NKG2C^−^ NK cells (Figure 7d).

Furthermore, NK cell degranulation was analyzed by assessing the surface level of CD107a in response to K562 target cells in some samples. Despite the high level of granzyme B, NK cells from patients with COVID-19 had lower degranulation activity as compared to healthy controls, suggesting dysfunctional cytotoxicity (Figure 7), while no difference in CD107a expression was observed between the recovered and lethal patients. At the same time, we found a positive correlation between the granzyme B level and CD107a expression in moderate-severity patients (Figure 7e). To test whether the low cytotoxic activity was related to NK cell exhaustion caused by a decrease in NKG2D expression and an increase in PD-1 expression, an appropriate correlation analysis was performed. A moderate positive correlation was found between the proportion of NKG2D^+^ NK cells and the degranulation level in the ICU patient group (Figure 7g). Moreover, the CD107a expression level had strong negative correlation with PD-1 expression in NK cells of the same donor group (r = −0.795; *p* = 0.01) (Figure 7f). A negative correlation was also established between the proportions of NKG2D^+^ and PD-1^+^ NK cells (Figure 5d). Thus, with a high degree of confidence, we can propose that PD-1 and NKG2D should be considered as markers of NK cell exhaustion during COVID-19 disease.

## 3. Discussion

COVID-19 causes a wide range of clinical manifestations, ranging from asymptomatic forms and standard symptoms of SARS to severe viral pneumonia and ARDS [3]. In severe forms of COVID-19, hyperactivation of the immune system occurs, primarily driven by the innate immune cells that mediate the excessive production of various cytokines and chemokines, causing a cytokine storm [49]. Our study is in agreement with previously published reports demonstrating the generalized dysregulation of both pro-inflammatory and anti-inflammatory cytokine production, which occurs already at the stage of moderate disease severity (Figure 3 and Figure S1). Lymphocyte characteristics during COVID-19 disease were addressed by many research groups, mostly reporting lymphopenia at the late stages of SARS-CoV-2 infection [9,10,12,27,38]. In this work, we not only confirmed a decrease in the proportion of CD45^high^CD14^−^ lymphocytes in the groups of ICU and moderate disease severity patients as compared to CCP and healthy donors, but also found that CD45^high^CD14^−^ cells were significantly lower in non-survivors as compared to the recovered ones among ICU patients (Figure 2). A statistically significant decrease in NK cell proportion was observed in the ICU patients as compared to other study groups, which also is in line with previous reports [8]. There may be several explanations for such a decrease in the NK cell population during moderate and severe COVID-19. These include the selective elimination of NK cells due to overactivation-induced apoptosis and platelet aggregation [50], as well as NK cell migration to the tissues, particularly to the lungs, where their SARS-CoV-2-induced accumulation has been shown [51]. In this work, we observed a high percentage of dying NK cells in the PBMC fraction of COVID-19 patients, with the highest values in severe COVID-19 patients (Figure 2c), which may also contribute to the ineffective immune control of viral replication [17].

In accordance with the already published data [2,10], we demonstrated a highly activated state of NK cells in both moderate and severe COVID-19 patient groups, which was determined by the increased expression level of HLA-DR, a well-known marker of NK cell activation (Figure 4a) [52]. In addition, we found an increase in the percentage of CD56^dim^ NK cells in the moderate disease severity group as compared to healthy controls (Figure 2b), which may be explained by the activation-mediated maturation of NK cells. An increase in the more differentiated NK cell pool has previously been reported in COVID-19 by other groups [50]. The activity of NK cells is highly dependent on the chemokines and cytokines produced by the microenvironment. Both the activation and differentiation of NK cells may be induced by high levels of IL-15 during COVID-19 (Figure 3e). IL-15 is vital for NK cell development and survival and mediates antiviral immunity [53]. In a cytokine profile study of COVID-19 patients, lower IL-15 levels distinguished non-survivors from survivors at a particular stage of the disease [54].

Another aspect of NK cell activation is their possible exhaustion. It is generally accepted that an inhibitory receptor, PD-1, demonstrates sustained expression in resting conventional T cells and overexpression upon their activation. This activation may occur as a result of antigen recognition during viral infections, and PD-1 mediates the prevention of an excessive response of protective immunity [55]. Concerning NK cells, PD-1 may possess a similar regulatory capacity. In healthy conditions, NK cells express PD-1 in trace amounts, in contrast to T cells [56]. The level of PD-1-positive NK cells was shown to be significantly higher in COVID-19 patients, and this phenotype was interpreted as the exhaustion of NK cells [17,57]. Our results confirm an increased percentage of PD-1-positive NK cells in ICU and moderate COVID-19 patients in comparison with healthy donors, accompanied by an elevated level of HLA-DR-expressing NK cells. Such an increase in PD-1 expression was observed both in the general population of NK cells and in the CD56^dim^ subset (Figure 4b). It has been previously shown that a higher PD-1 expression level implies a lower degranulation capacity of NK cells [19]. The strong negative correlation between PD-1 expression and the NK cell degranulation level, which we found in the ICU patient group, supports this notion (Figure 7a). Indeed, PD-1 surface expression should be considered as evidence of NK cell exhaustion.

NK-cell-mediated target elimination is based on a balance between activation and inhibition signals depending on the engagement of multiple activating and inhibitory receptors. The NK-cell-activating receptors, such as NKG2D and CD16, are involved in the interaction with SARS-CoV-2-infected cells. A decrease in the NKG2D expression level in NK cells was reported in COVID-19 [27]. Furthermore, increased circulating levels of IL-6 may contribute to low NKG2D expression [58]. A proteomic analysis showed that NKG2D ligands such as MICA and ULBP2 decrease during SARS-CoV-2 infection in cell line models [59]. On the other hand, an increase in the soluble MICA concentration in plasma samples obtained from COVID-19 patients compared to healthy volunteers was also reported [60]. The hyperactivation of NKG2D^+^ NK cells via soluble stress molecules may cause the impaired functional response of NK cells [61]. Interestingly, NKG2D is able to interact with a SARS-CoV-2-derived peptide, which influences NK cells’ activation [62].

NK cell ADCC apparently plays an important role in the COVID-19 immune response [63]. Expression of CD16 is decreased during acute COVID-19 [17,64]. It was noticed that patients with the high-affinity allele of the CD16 receptor were more susceptible to hospitalization during COVID-19 disease. Moreover, 50% of the patients with lethal outcomes were homozygous for the high-affinity allele [65]. We showed that the NKG2D^+^ and CD16^+^ NK cell subsets were decreased in ICU patients as compared to healthy donors. Additionally, we found that the decrease in CD16^+^ and NKG2D^+^ NK cells is a coordinated process in COVID-19 patients. Furthermore, ICU patients with lethal outcomes had significantly less NKG2D^+^ NK cells, and there was a tendency towards a decrease in CD16^+^ NK cell levels in samples from these patients (Figure 5). Taken together, our data reveal that NK cells from ICU patients with lethal outcomes are characterized by the loss of NKG2D- and CD16-activating receptor expression on their surface.

Substantial changes in the antiviral NK cell response may occur via alterations in the surface expression of the inhibitory receptors, such as NKG2A and KIR2DL2/DL3. In an early work describing immune cells in COVID-19, an increase in the NKG2A expression level was reported [66]. Increased expression of NKG2A by NK cells in COVID-19 was considered as a signal of the depletion and inhibition of the antiviral immune response [8]. It was shown that NK cells expressing NKG2A were activated in patients with COVID-19 due to the interactions of the NKG2A receptors with the HLA-E molecules, presenting a SARS-CoV-2 Nsp13-encoded peptide. These interactions abrogated NKG2A-mediated NK cell inhibition and were able to restrain SARS-CoV-2 replication in vitro [67]. In our work, we did not find statistically significant differences in NKG2A expression levels between the studied groups (Figure 5). We also observed no differences in the expression of inhibitory KIR2DL2/DL3 receptors on NK cells, both in the COVID-19 patients and healthy donors. Our data are consistent with those obtained by other groups [10,11].

The role of the so-called adaptive NK cells in the development of COVID-19—in particular, the effect of the long-lived adaptive NK cells associated with HCMV, which are present in the blood of a significant proportion of healthy HCMV-seropositive people—remains insufficiently studied. The involvement of this NK cell pool, often expressing NKG2C [34], in the pathogenesis of COVID-19 is difficult to study without knowing the size and properties of this cell subset prior to the COVID-19 disease. It was previously shown that an increased proportion of highly differentiated NK cells with a reduced FcεRγ expression level distinguished lethal patients from those who recovered [26]. Later, it was reported that FcεRγ-negative NK cells are characterized by reduced proliferative activity [36]. According to our data, a decreased NKp30 expression level also differentiated NK cells among samples from non-survivors and recovered patients in the severe COVID-19 group. With a high degree of certainty, we describe the same subset of adaptive NK cells that have undergone significant epigenetic changes. The absence of any correlations between the proportions of NKp30^+^, CD57^+^, and NKG2C^+^ NK cells in the group of severe COVID-19 patients evidenced that the adaptive-like NK cell subset had apparently not formed during the disease. It is unclear whether NK cells expressing NKG2C are an aggravating factor in COVID-19 pathogenesis. On the one hand, the absence of the KLRC2 gene encoding NKG2C has been shown to increase the risk of severe COVID-19 [37]. On the other hand, Maucourant et al. reported an increased level of NKG2C^+^CD57^+^CD56^dim^ NK cells in patients with severe COVID-19, although the cohort in their study included only 17 patients [10]. In another study, the level of NKG2C expression did not correlate with the level of FcεRIγ expression [36]. In our work, a reduced proportion of NKp30^+^ NK cells in the COVID-19 patients with lethal outcomes was not accompanied by an increase in NKG2C expression. Rather, a trend towards a decreased proportion of NKG2C^+^ NK cells in non-survivors compared to the recovered patients was observed (Figure 6). Moreover, higher content of NKG2C^+^ NK cells was recorded in the group of moderate patients as compared to other groups. Additional correlations found between the proportions of NKG2C^+^, CD57^+^, and NKp30^+^ NK cells in moderate patients and convalescents may indicate the coordinated expression of these molecules and the formation of a NKG2C^+^CD57^+^NKp30^−^ NK cell pool. We previously showed that the less differentiated NKG2C^+^ precursors of the adaptive NK cells have increased proliferative potential [68]. A multi-omics study found a proliferating subset of NK cells increasing from mild to moderate COVID-19 disease, reflecting NK cell activation and differentiation in response to the SARS-CoV-2 virus [69]. It can be suggested that a proliferative response involving NKG2C^+^ NK cells, actively producing granzyme B (Figure 7d), may play a positive role in COVID-19 outcome. Higher content of granzyme B in NKG2C^+^ NK cells was shown in our previous work in a healthy cohort [68]. It should be also noted that the SARS-CoV-2 Spike protein is supposed to be involved in the overexpression of the HLA-E molecule in the immune and stromal cells in the bronchoalveolar lavage fluids of COVID-19 patients [70]. HLA-E is the ligand for both NKG2A and NKG2C receptors; thus, further studies of SARS-CoV-2-specific interactions of these receptors with their ligands are needed.

The cytotoxic activity of NK cells is their essential characteristic, which reflects the cell activation state. An elevated level of granzyme B is usually considered as evidence of the maturity of NK cells and their ability to induce cytotoxicity. An increase in the proportion of granzyme-B-expressing NK cells during COVID-19 has been reported in several studies [10,71]. We also observed higher levels of granzyme B^+^ NK cells in the ICU patient group compared to moderate-severity COVID-19 patients and CCP donors (Figure 7). The increase in granzyme B levels in NK cells may be partially mediated by pro-inflammatory cytokines actively produced during COVID-19, such as IL-15.

It was reported earlier that, despite the elevated intracellular concentrations of granzyme B, NK cells in COVID-19 are cytotoxically dysfunctional [15]. In our study, we also observed significantly decreased K562-induced degranulation in NK cells from COVID-19 patients (Figure 7). A decreased NKG2D level in both ICU and moderate disease severity groups, and correlations found between the degranulation activity, NKG2D, and PD-1 expression levels, strongly support the idea that NKG2D loss is involved in the natural cytotoxicity failure and the exhausted state of NK cells during COVID-19. Dysfunction of NK cells may be also caused by the increased production of anti-inflammatory cytokines, such as TGFβ, during COVID-19 [7].

**Figure 8:**
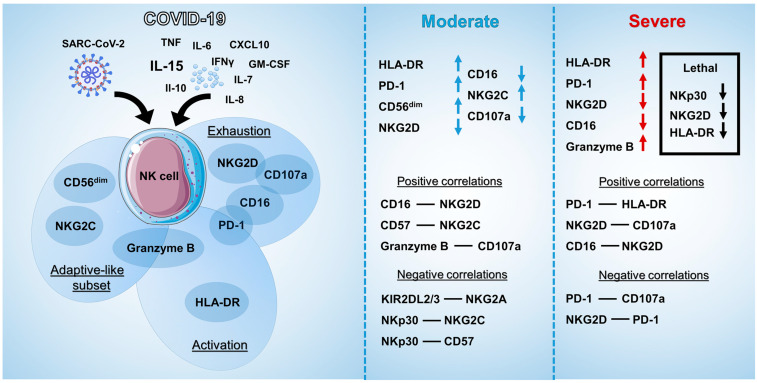
Scheme summarizing the results obtained in the work. Left part represents the main effects of SARS-CoV-2 and cytokines on NK cells’ phenotype and functional activity (adaptive-like subset, activation, and exhaustion). Middle and right parts represent the results of the study, where the significant changes in NK cell markers are highlighted by arrows, and positive and negative correlations are listed for moderate and severe COVID-19 patients.

Taken together, and in line with previously published data, we demonstrate the coordinated loss of activation receptors and acquisition of exhaustion- and adaptivity-associated markers by NK cells accompanied by generalized cytokine dysregulation in COVID-19 patients, as summarized in Figure 8.

## 4. Materials and Methods

### 4.1. Patient and Donor Characteristics

The collection of blood samples from patients and healthy donors was carried out based on the Federal Research and Clinical Center for Specialized Types of Medical Care and Medical Technologies (FMBA). Written informed consent to participate in the study was received from each individual.

Criteria for the inclusion of a patient in the study: the presence of SARS-CoV-2 infection, confirmed by PCR examination of a sample obtained from the upper respiratory tract and/or by clinical and radiological symptoms (presence of a characteristic clinical picture and characteristic signs of polysegmental viral pneumonia, corresponding to the classification of CT2-CT3-CT4 COVID-19). Saturation level SpO2 ≤ 93%, being on intermittent mandatory ventilation (IMV), treatment in the pulmonology department of the Federal Scientific and Practical Center of the Federal Medical and Biological Agency, the absence or presence of unfavorable factors for a severe course of disease (diabetes mellitus, obesity, CVD, and other comorbid conditions that worsen the probability of recovery) did not prevent patients from being included in the study. The criterion for exclusion from the study was the presence of either a blood disease or an oncological disease.

The following comparison groups were formed: (1) 34 COVID-19 patients staying in the intensive care unit (ICU patients) (aged from 30 to 82 years, median age 61 years; SD ± 11.5; 60% male); (2) 28 patients with moderate COVID-19 (moderate severity) (aged from 23 to 93 years, median age 59 years; SD ± 17.5; 64% male); (3) 33 COVID-19 convalescent plasma donors (CCP donors), recovered from COVID-19 less than a month previously, with a high SARS-CoV-2-specific antibody titer in plasma (aged from 33 to 54 years, median age 45 years; SD ± 6.8; 60% male); (4) 48 healthy donors—a group of volunteers who had not suffered COVID-19 in the past 6 months (aged from 28 to 73 years, median age 40 years; SD ± 14.3; 41% male).

### 4.2. Sample Collection

To isolate the peripheral blood mononuclear cells (PBMC), blood samples were collected in EDTA-containing test tubes and centrifuged in a Ficoll gradient with a density of 1.077 g/cm^3^. Plasma samples were collected and stored at −80 °C until further analysis. Obtained PBMC samples were immediately analyzed by flow cytometry.

### 4.3. Phenotype and Viability Analysis

PBMC samples were stained with the fluorochrome-conjugated monoclonal antibodies, which are shown in Supplementary Table S1. The following mouse anti-human fluorescent-labeled antibodies were used for surface cell staining: NKG2C-FITC (clone REA205, Miltenyi Biotec, Bergisch Gladbach, Germany), HLA-DR-PE (clone L243, Sony Biotechnology, San Jose, CA, USA), CD3-PerCP (clone HIT3a, Sony Biotechnology San Jose, CA, USA), CD3-FITC (clone FIT3a, Sony Biotechnology, San Jose, CA, USA), CD3-Vioblue (clone BW264/56, Miltenyi Biotec, Bergisch Gladbach, Germany), CD56-APC-Vio770 (clone REA196, Miltenyi Biotec, Bergisch Gladbach, Germany), CD56-APC (clone N901, Beckman Coulter, Brea, CA, USA), KIR2DL2/L3-APC (clone DX27, Sony Biotechnology, San Jose, CA, USA), CD45-VioBlue (clone REA747 Miltenyi Biotec, Bergisch Gladbach, Germany), CD57-APC (clone HNK-1, Sony Biotechnology, San Jose, CA, USA), CD14-PE-Cy7 (Miltenyi Biotec, Bergisch Gladbach, Germany), NKp30-PE (clone P30-15, Sony Biotechnology, San Jose, CA, USA), NKG2D-PE (clone 1D11, Sony, Sony Biotechnology, San Jose, CA, USA), CD45-PerCP (clone 2D1, Sony Biotechnology, San Jose, CA, USA), CD16-APC (Miltenyi Biotec, Bergisch Gladbach, Germany), PD-1-Alexa Fluor 647 (clone EH12.2H7, BioLegend, San Diego, CA, USA). FMO controls were performed for mouse anti-human fluorescent-labeled antibodies (Figure S3 and Figure 7c). In addition to monoclonal antibodies, staining with SytoxBlue Dead Cell stain was performed (Invitrogen, Waltham, MA, USA).

Samples were analyzed using a MACSQuant 10 flow cytometer (Miltenyi Biotec, Bergisch Gladbach, Germany) equipped with lasers λ = 405 nm, λ = 488 nm, λ = 635 nm; threshold was set to cut-off events with low CD45 staining.

### 4.4. Granzyme B Staining

PBMC samples were fixed with a kit for cell fixation and permeabilization (Miltenyi Biotec, Bergisch Gladbach, Germany); after this, intracellular staining with Alexa Fluor 647-labeled antibodies to granzyme B (clone GB11, BioLegent, San Diego, CA, USA) was performed. Cells were analyzed by flow cytometry.

### 4.5. Functional Test

Analysis of the natural cytotoxic activity of NK cells was conducted in PBMC samples by estimation of the NK cell degranulation level by flow cytometry. PMBCs were stimulated overnight with IL-2, 500 U/mL (Sci-Store, Moscow, Russia). Degranulation was determined by the level of expression of the lysosomal marker LAMP-1 (CD107a) using CD107a-VioBlue monoclonal antibody (clone REA792, Miltenyi Biotec, Bergisch Gladbach, Germany), as described earlier [71]. To prevent fluorescence inactivation due to the reuptake of vesicles containing LAMP-1 molecules, 10 mg/mL monensin was used (ICN Biomedical, Erie, PA, USA), which blocks intracellular transport from the endoplasmic reticulum to the Golgi apparatus. K562 target cells were added to NK cells in a 1:1 ratio. After 4 h of incubation, cells were labeled with fluorescent-labeled monoclonal antibodies to CD14, CD45, CD56, CD3, NKG2C and analyzed by flow cytometry. NK cells were identified as CD3^−^CD56^+^ cells.

### 4.6. Cytokine Production

Unbiased analysis of serum cytokine production was performed using Luminex xMAP multiplex technology and the MILLIPLEX MAP Human Cytokine/Chemokine Magnetic Bead Panel Kit, according to the manufacturer’s standard protocol (Merck, Rahway, NJ, USA). Data processing was carried out using Belysa software v1.1.0 (Merck, Rahway, NJ, USA) at the Resource Center “Cell Technology and Immunology”, Sirius University of Science and Technology.

### 4.7. Statistical Analysis

The data were analyzed using FlowJo X 10.0.7r2 (FlowJo LLC, Ashland, OR, USA) and GraphPad Prism 8.00 software (StatSoft Inc., Tulsa, OK, USA) and presented as mean ± standard deviation. Statistical analysis for parametric samples was carried out using the ANOVA test for multiple comparisons; Kruskal–Wallis test was used for nonparametric samples. For paired data, paired two-sample Student *t*-test or the Wilcoxon signed rank test were used. The value of *p* < 0.05 was considered statistically significant.

## Data Availability

Data are available on request.

## Supplementary Materials

The following supporting information can be downloaded at: https://www.mdpi.com/article/10.3390/ijms24031996/s1.
