# Supplementary material for: Coordinated Loss and Acquisition of NK Cell Surface Markers Accompanied by Generalized Cytokine Dysregulation in COVID-19
Source: Int J Mol Sci. 2023 Jan 19;24(3):1996. doi: 10.3390/ijms24031996 (PMC9917026; doi:10.3390/ijms24031996)
Supplement: Supplementary file 1 [file ijms-24-01996-s001.zip › ijms-2102382-supplementary.pdf]

Supplementary Table S1. The panels of fluorochrome-conjugated monoclonal antibodies, which were used in the study.

	**Panel 1**	**Panel 2**	**Panel 3**	**Panel 4**	**Panel 5**	**Panel 6**
VioBlue	CD3	CD3		CD3	CD45	CD107a
FITC	NKG2C		CD3		NKG2C	CD3
PE	NKG2A	NKp30	NKG2D	HLA-DR		
PerCP	CD45	CD45	CD45	CD45	CD3	CD45
PE-Cy7	CD14	CD14	CD14	CD14	CD14	CD14
APC	CD57	KIR2DL2/3	CD16	PD-1	Granzyme B	CD56
APC-Vio770	CD56	CD56	CD56	CD56	CD56	

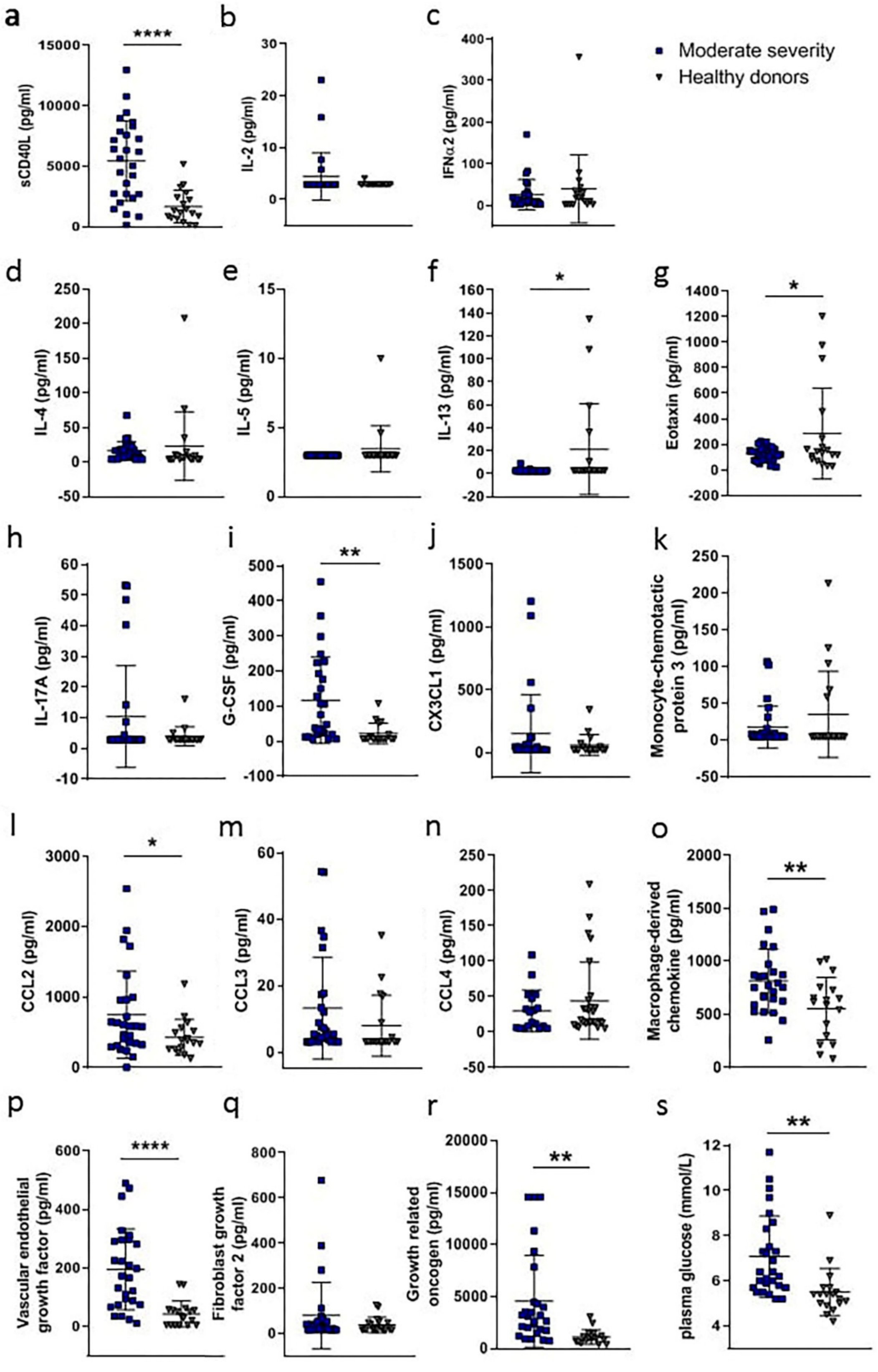

Supplementary Figure S1. Concentration of (a) sCD40L, (b) IL-2, (c) IFNα2, (d) IL-4, (e) IL-5, (f) IL-13, (g) Eotaxin, (h) IL-17A, (i) G-CSF, (j) CX3CL1, (k) MCP3, (l) CCL2, (m) CCL3, (n) CCL4, (o) MDC, (p) VEGF, (q) FGF2, (r) GRO in pg/ml in serum of healthy donors and patients with moderate COVID-19 measured with MagPix assay. (s) concentration of plasma glucose (mmol/L) measured with glucometer. *p<0.05, **p<0.01,****p< 0.0001.

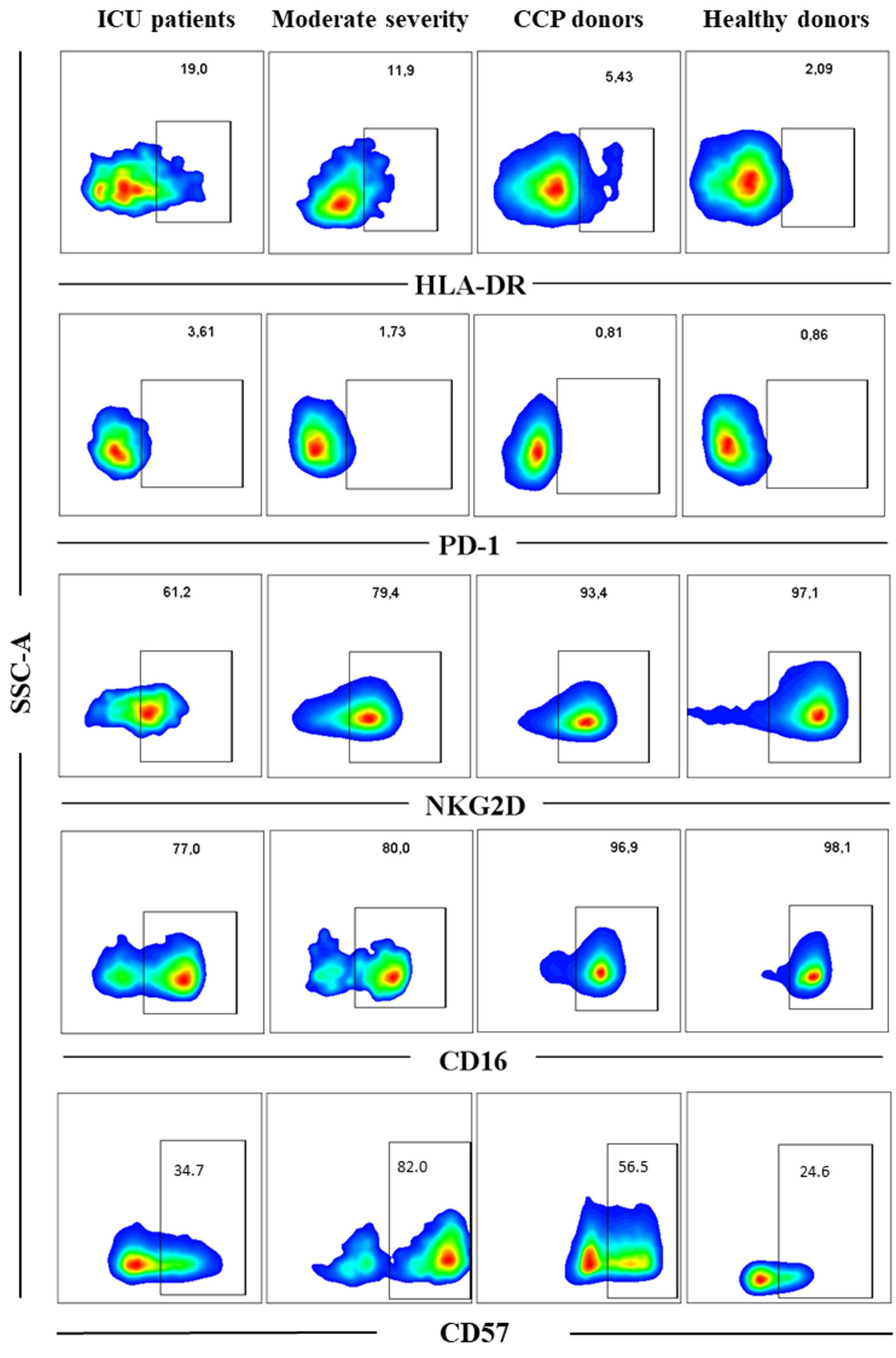

Supplementary Figure S2. Representative expression of phenotypic markers (HLA-DR, PD-1, NKG2D, CD16, CD57) on NK cells from ICU patients, moderate severity patients, CCP donors and healthy donors.

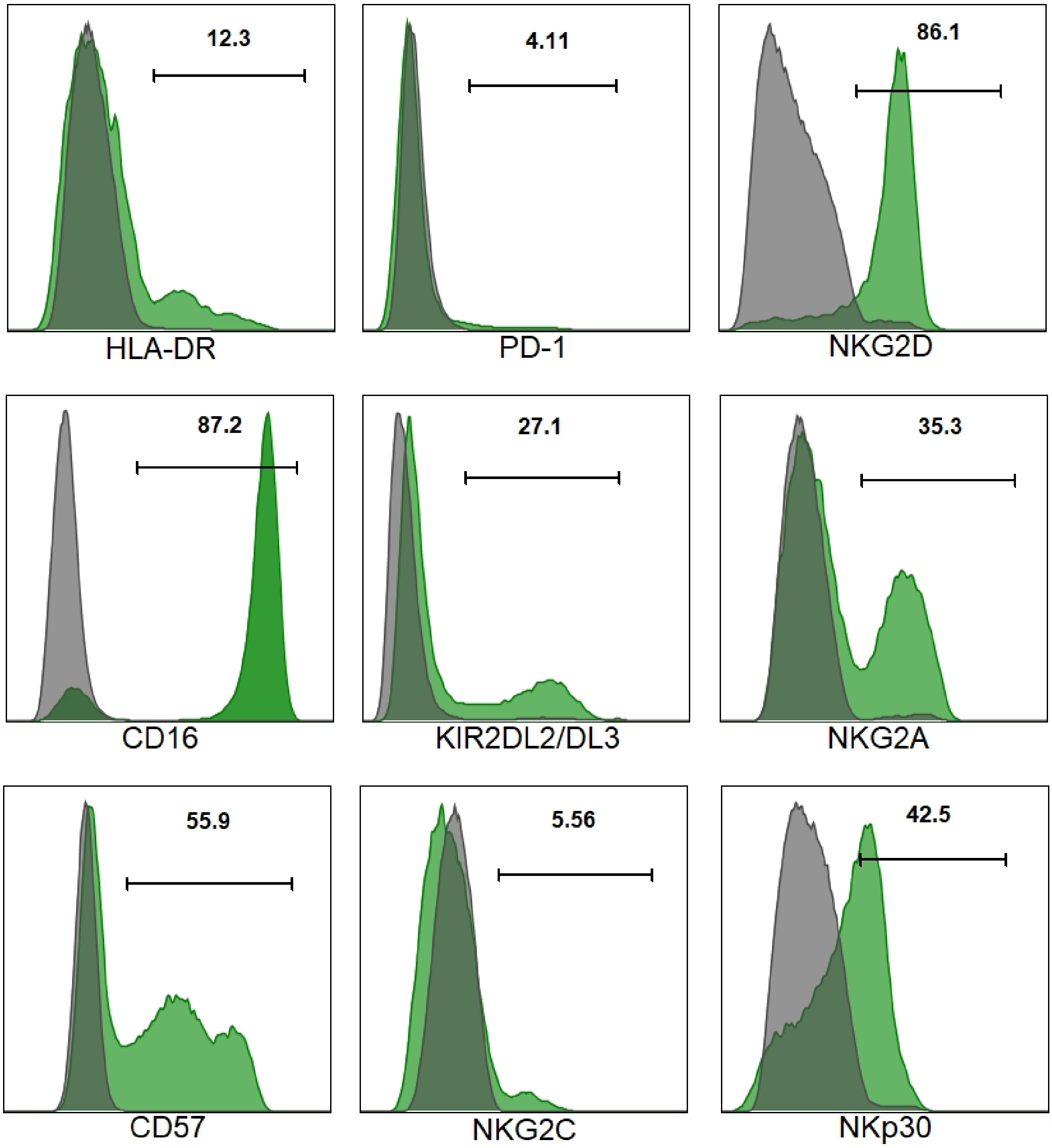

Supplementary Figure S3. FMO (fluorescence minus one) controls (grey) presented as a histogram for HLA-DR-PE, PD-1-Alexa Flour 647, NKG2D-PE, CD16-APC, KIR2DL2/DL3-APC, NKG2A-PE, CD57-APC, NKG2C-FITC, and NKp30-PE (green).
